# Supplementary material for: Fatty Acid Amides Suppress Proliferation via Cannabinoid Receptors and Promote the Apoptosis of C6 Glioma Cells in Association with Akt Signaling Pathway Inhibition
Source: Pharmaceuticals (Basel). 2024 Jul 2;17(7):873. doi: 10.3390/ph17070873 (PMC11280372; doi:10.3390/ph17070873)
Supplement: Supplementary file 1 [file pharmaceuticals-17-00873-s001.zip › pharmaceuticals-2990214-supplementary.pdf]

Supplementary figures

Fatty Acid Amides Suppress Proliferation via Cannabinoid Receptors and Promote the Apoptosis of C6 Glioma Cells in Association with Akt Signaling Pathway Inhibition

Nágila Monteiro da Silva, Izabella Carla Silva Lopes, Adan Jesus Galué-Parra, Irlon Maciel Ferreira, Chubert Bernardo Castro de Sena, Edilene Oliveira da Silva, Barbarella de Matos Macchi, Fábio Rodrigues de Oliveira and José Luiz Martins do Nascimento *

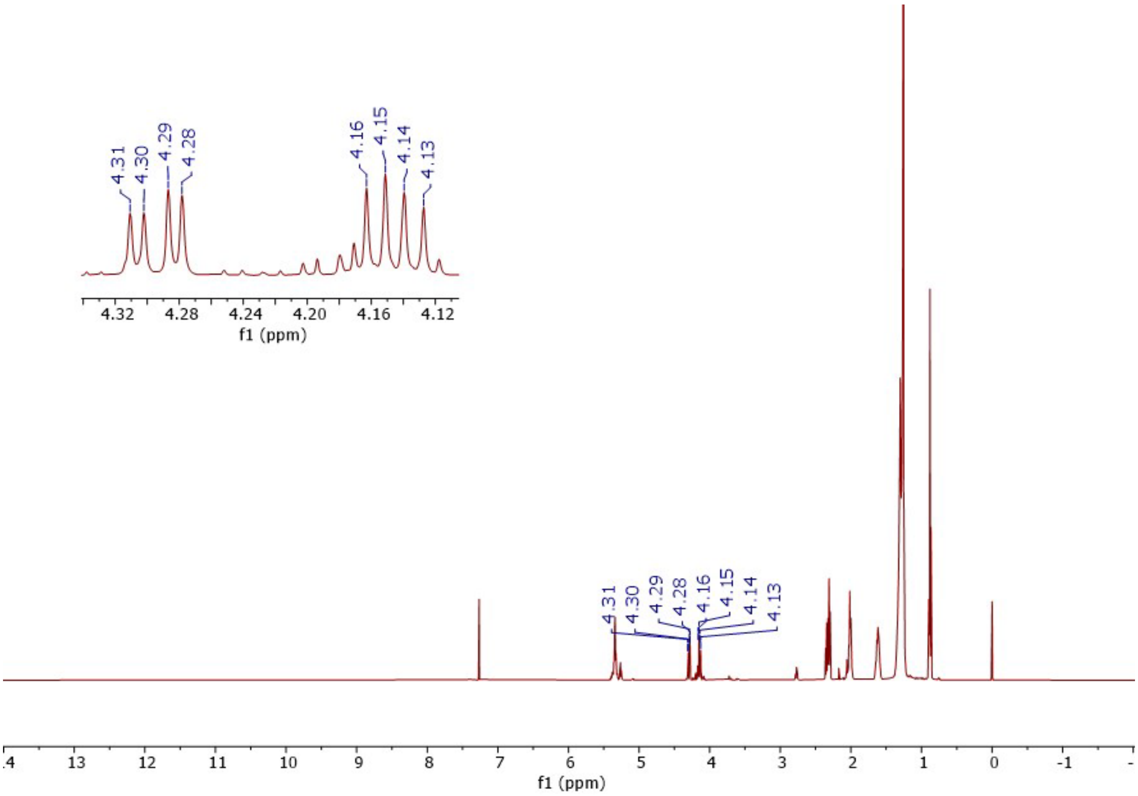

Figure S1: ^{1}H NMR spectrum of AO1.

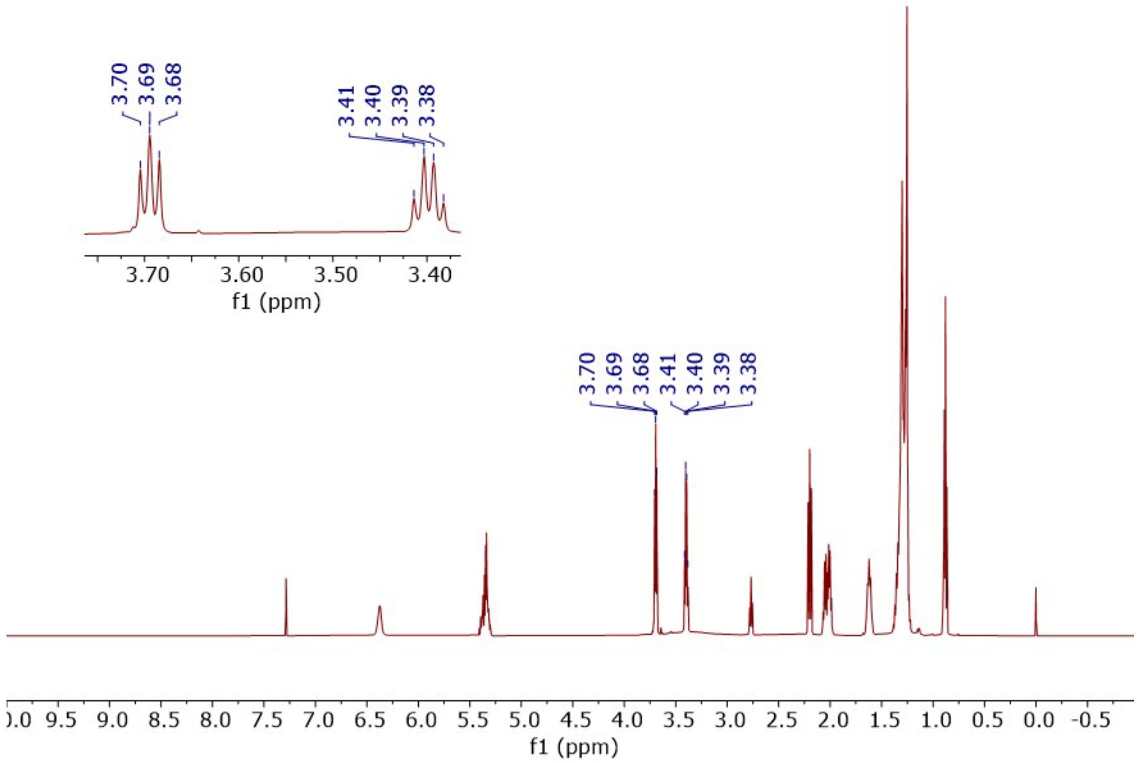

Figure S2: ^{1}H NMR spectrum of FAA1.

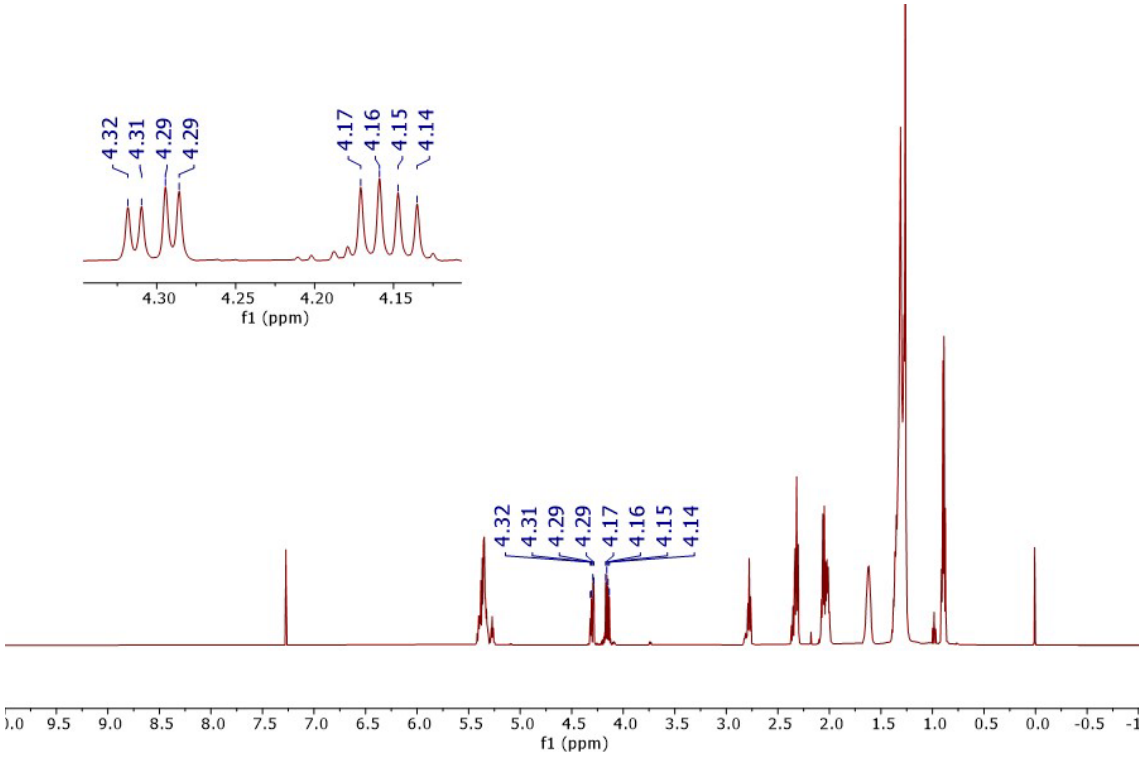

Figure S3: ^{1}H NMR spectrum of AO2.

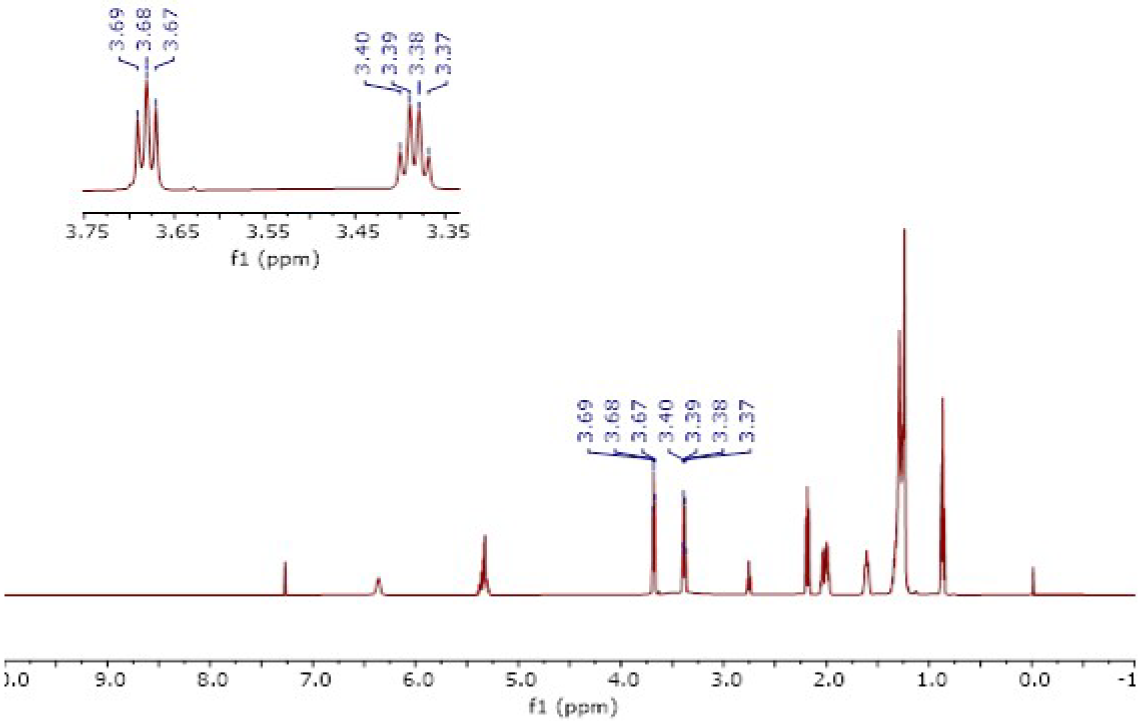

Figure S4: ^1H NMR spectrum of FAA2.
